# Influence of the Nd:YAG Laser Pulse Duration on the Temperature of Primary Enamel

**DOI:** 10.1155/2015/396962

**Published:** 2015-03-22

**Authors:** R. A. Valério, V. S. da Cunha, R. Galo, F. A. de Lima, L. Bachmann, S. A. M. Corona, M. C. Borsatto

**Affiliations:** ^1^Clinical Pediatric Dentistry Department, Ribeirão Preto School of Dentistry, São Paulo University, Café Avenue, Monte Alegre, 14040-904 Ribeirão Preto, SP, Brazil; ^2^Physics Department, School of Philosophy, Science and Literature, São Paulo University, 3900 Bandeirantes Avenue, 14040-901 Ribeirão Preto, SP, Brazil; ^3^Restorative Dentistry Department, Ribeirão Preto School of Dentistry, São Paulo University, Café Avenue, Monte Alegre, 14040-904 Ribeirão Preto, SP, Brazil

## Abstract

The aim of this study is to evaluate the temperature change on specimens of primary enamel irradiated with different pulse duration of Nd:YAG laser. Fifteen sound primary molars were sectioned mesiodistally, resulting in 30 specimens (3.5 × 3.5 × 2.0 mm). Two small holes were made on the dentin surface in which K-type thermocouples were installed to evaluate thermal changes. Specimens were randomly assigned in 3 groups (*n* = 10): A = EL (extra long pulse, 10.000 *μ*s), B = LP (long pulse, 700 *μ*s), and C = SP (short pulse, 350 *μ*s). Nd:YAG laser (*λ* = 1.064 *μ*m) was applied at contact mode (10 Hz, 0.8 W, 80 mJ) and energy density of 0.637 mJ/mm^2^. Analysis of variance (ANOVA) was performed for the statistical analysis (*P* = 0.46). Nd:YAG laser pulse duration provided no difference on the temperature changes on primary enamel, in which the following means were observed: A = EL (23.15°C ± 7.75), B = LP (27.33°C ± 11.32), and C = SP (26.91°C ± 12.85). It can be concluded that the duration of the laser pulse Nd:YAG increased the temperature of the primary enamel but was not influenced by different pulse durations used in the irradiation.

## 1. Introduction

Developed by Johnson in 1961 [1], the Nd:YAG (neodymium doped with yttrium-aluminum-garnet, *λ* = 1.064 *μ*m) can be employed at continuous or pulsed mode emitting light with a wavelength located in the infrared range of the electromagnetic spectrum. This high-intensity laser usually has the diode laser as light guide and its absorption is diffuse and transmitted to the tissue through the optical fiber, favoring its application on the buccal cavity [2].

The Nd:YAG laser can be recommended in pediatric dentistry, since its use promotes increasing on the acid resistance of primary enamel [3, 4], sealing of pits and fissures [5] and effectiveness on the prevention of carious lesions [6, 7]. The parameters used during irradiation of dental structures must comply with the characteristics of the tissues, since the variation in surface temperature of the irradiated enamel can lead to higher heat conduction and hence spread to the pulp tissue causing irreversible damage. The temperature inside the pulp chamber should not exceed 5.5°C [8], since such heating may lead to tooth loss of vitality.

The thermal effect produced by Nd:YAG irradiation induces the formation of TCPCa_3_(PO_4_)^2^ on enamel [9] and promotes changes in the organic matrix [9, 10] and alterations in the morphological [11], chemical [12, 13] and crystallographic aspects [14]. In dentin, the thermal effect promotes melting, formation of cracks and debris on the surface, and the modification of tubular dentin structure [15]. These alterations on dentin can occur due to the lower thermal conductivity of this substrate when compared to the primary enamel [16–18].

Thermal variations after employing Nd:YAG laser in permanent teeth have been reported [19–21]. The thermal changes on the pulp chamber of primary teeth, caused by the Nd:YAG laser (picosecond-pulsed), were verified by Lizarelli et al., 2006 [18], using ablative parameters. The Nd:YAG laser (picosecond-pulsed) was considered by Lizarelli et al., 2006 [18], as a safe tool for primary teeth ablation, after checking the temperature response in the pulpal chamber and the anatomical constitution of teeth, once different topography of teeth results in profound differences in remaining dentin, with consequences for heat exchanges rate.

The pulse duration refers to the time that the tooth substrate is exposed to laser irradiation. The irradiation of primary teeth substrate promotes different times of thermal declines, which are related to the anatomical features [18]. The surface cooling can also be performed reducing the pulse duration [22], whereas the emission of shorter pulses can cause no thermal damage [23] to the irradiated surfaces. The pulse repetition rate has been described as being an important parameter with respect to heat deposition on lased-irradiated tissue. The higher the pulse repetition rate, the lower the cooling of the tissue between each pulse [24].

Due to the increased research, related to the use of laser technology in pediatric dentistry, and regarding the heat generation produced on dental substrates during irradiation, more studies evaluating the thermal changes in specimens of primary teeth irradiated with Nd:YAG laser using different pulse duration, extra long pulse (10.000 *μ*s), long pulse (700 *μ*s), and short pulse (350 *μ*s), become necessary.

## 2. Material and Method

### 2.1. Experimental Design

The factor studied was the Nd:YAG laser pulse duration employed during the irradiation of enamel specimens of primary molars at 3 levels: A = EL (extra long pulse, 10.000 *μ*s), B = LP (long pulse, 700 *μ*s), and C = SP (short pulse, 350 *μ*s). The experimental sample was composed by 30 specimens of primary human enamel, which were randomly assigned (*n* = 10), according to the design in randomized complete blocks. The quantitative response variable was the temperature change, in Celsius degrees, of the primary tooth substrate subjected to the Nd:YAG laser irradiation.

### 2.2. Teeth Selection and Preparation of Specimens

First and second upper primary human molars newly exfoliated were examined with an explorer probe #5 (Duflex, SSWhite, Rio de Janeiro, RJ, Brazil), using a stereomicroscope (Leica S6 D Stereozoom, Mycrosystems Leica AG, Switzerland), with increase of 20x. Those which presented cracks or hypoplasia were discarded. Fifteen teeth were selected and cleaned with periodontal curettes, being polished with Robinson brushes mounted in low speed turbine (Dabi Atlante, Ribeirão Preto, São Paulo, SP, Brazil) embedded in pumice and water, washed, and kept in 0.9% saline solution containing 0.4% sodium azide at 4°C [25].

The teeth were individually fixed by the coronary portion with thermoplastic wax (Wax Sculpture Fixed Prosthodontics, Aspheric Chemical Industry Ltda., São Caetano do Sul, São Paulo, SP, Brazil) in acrylic plates and taken to the section machine (Minitom, Struers A/S, Copenhagen, Denmark) in which the root portion, if it is present, was sectioned 1 mm below the cement-enamel junction. Then, the coronary portions were sectioned mesiodistally and from the buccal and lingual surfaces of each tooth specimens of 3.5 × 3.5 × 2.0 mm of thickness of enamel and dentin were obtained. The dimensions of the specimens were determined using a digital caliper (Myamoto, Tokyo, Japan), and the thickness was established with a specimeter (BioArt, São Carlos, São Paulo, SP, Brazil).

To standardize the thickness of enamel/dentin, specimens were taken to the polisher (Politriz DP-9U2, Struers A/S, Copenhagen, Denmark) and subjected to wear with sanding discs of silicon carbide #600 (Norton/Saint-Gobain Abrasivos Ltda., Guarulhos, São Paulo, SP, Brazil), aiming at planning and regularizing the surfaces. Using drill #1/4 (KG Sorensen, Barueri, São Paulo, SP, Brazil), mounted in high speed turbine (Roll Air 3, Kavo do Brasil S.A, Joinville, Santa Catarina, SC, Brazil), two holes were made manually by the same operator, at dentin surface (medium depth 0.1 mm) corresponding to the roof of the pulp chamber, to accommodate the thermal-sensors during the specimens irradiation. After these procedures, specimens were individually fixed with thermoplastic wax at cylindrical Plexiglass abutment (5.0 mm diameter) using a parallelometer to ensure that the enamel surface was kept parallel to the horizontal plane, aiming at sealing any space between the specimen and the plaque. The specimens were randomly assigned (*n* = 10) and kept in distilled water at 4°C until 2 hours before the experiment start [26].

The irradiated area was delimited by insulating type (3M do Brasil Ltda., Campinas, São Paulo, SP, Brazil), with a 4.0 mm^2^ central window. Each group was irradiated with Nd:YAG laser (*λ* 1.064 *μ*m) (Smartfile, Deka M.E.L.A, Calenzano, Firenze, Italy), at contact mode by means of 0.3 mm quartz fiber, which was positioned perpendicularly to the specimen. The parameters (10 Hz, 0.8 W, 80 mJ), energy density of 0.637 mJ/mm^2^, and different pulse duration: A = EL (extra long pulse, 10.000 *μ*s), B = LP (long pulse, 700 *μ*s), and C = SP (short pulse, 350 *μ*s), were applied to the specimens of primary enamel, for 30 seconds.

The laser parameters of Nd:YAG (10 Hz, 0.8 W, 80 mJ) used in this study were based on the favorable results obtained by [4, 6, 27, 28], to increase the acidic resistance to demineralization.

To evaluate the temperature change, a device consisting of a data acquisition card HI-Speed USB Carrier NI USB-9162 (National Instruments Corporation, Austin, Tx, USA) was used. The thermal filaments sensors (K-type thermocouples, Omega Engineering Inc., USA) were used to check the temperature. The board has 4 input channels with full resolution of 24 bits and 12 Hz maximum total rate of data acquisition.

The thermocouples were made with the aid of a spot welding of carbon and presented 120 *μ*m of diameter and 40 cm in length. The system was connected to a computer and the Measurement and Automation Software and VI Logger Lite (National Instruments Corporation, Austin, Tx, USA), supplied by the manufacturer of data acquisition card, was employed for data collection. Two thermal-sensors strands were placed in the niches at the dentin surface of each specimen, providing better thermal contact between the thermal-sensors and the specimens; a thermal paste based on water was employed (Implastec, Votorantim, São Paulo, SP, Brazil).

**Figure 1:**
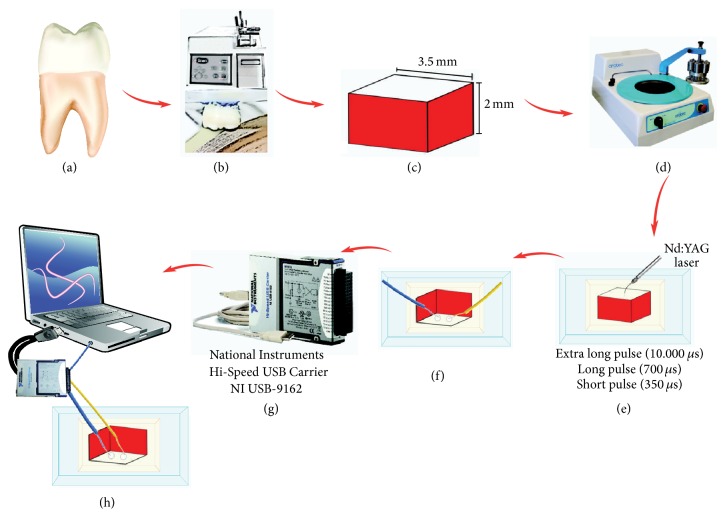
Schematic design of the employed methodology. (a) Primary molar; (b) section machine and sectioned tooth; (c) specimens (3.5 × 3.5 × 2.0 mm); (d) polisher; (e) fixation of specimens at acrylic plates, delimitation of irradiated area with insulating tape (4.0 mm^2^), and irradiation with Nd:YAG laser; (f) holes to accommodate thermal-sensors (0.1 mm depth); (g) acquisition plate; (h) system connected to the computer.

For each specimen, the temperature (°C) was registered from the first laser pulse emitted by the Nd:YAG laser and repeated every 0.3 seconds, for 30 seconds using the K-type thermocouples adapted to the dentin surface 1 mm, under irradiated surface, and all measurements were performed in a temperature/humidity-controlled room.  Figure 1 represents the schematic design of the employed methodology.

### 2.3. Statistical Analysis

The temperature mean value of each specimen was analyzed using the Kolmogorov-Smirnov test and presenting normal distribution and homogeneity of variance. Thus, analysis of variance (ANOVA) was used. Statistical analysis was performed with SPSS software for Windows, version 12.0 (SPSS Inc., Chicago, IL, USA).

## 3. Results

Results showed that temperature change during Nd:YAG laser irradiation using different pulse duration in specimens of primary enamel was statistically similar among themselves (*P* = 0.46), as shown in Table 1.

**Figure 2:**
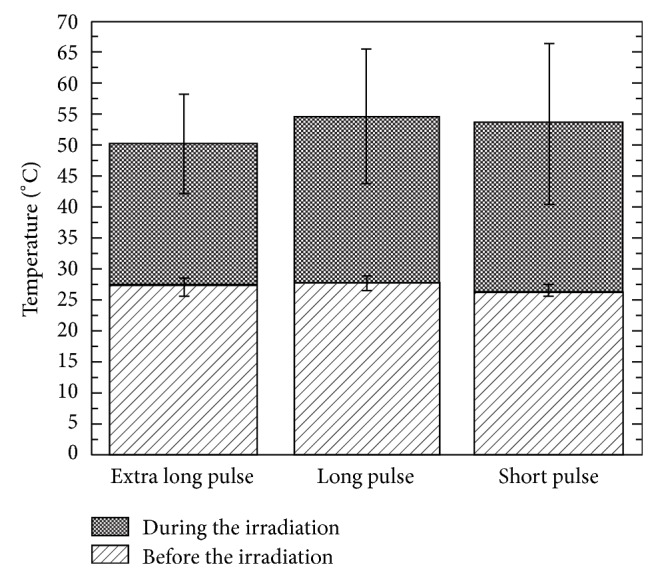
Initial temperature analyzed before irradiation and thermal changes (°C) measured at specimens of primary enamel during the Nd:YAG laser irradiation.

The initial temperature and thermal changes during Nd:YAG laser irradiation at specimens of primary enamel are shown in Figure 2.

## 4. Discussion

The utilization of laser light to irradiate dental tissues has aroused great interest in the scientific community. The effects of laser irradiation on the tissue depend on the parameters used, such as wavelength, power, power density, exposure time, pulse duration, emission mode, energy density used per pulse, repetition rate, frequency, diameter, and beam characteristics [29, 30]. The use of reliable parameters for irradiation on dental structures may prevent possible thermal damage which could lead to irreversible pulp damage.

The increasing in temperature on dental tissues, produced during irradiation, is responsible for the morphological and structural changes of the irradiated surface [31, 32], thus making the tooth enamel acid resistant [29, 30].

According to Fowler and Kuroda, 1986 [12], increases in dental enamel temperatures result in structural and chemical alterations, such as loss of water and reduction in carbonate content. Besides, acid phosphate (HPO_4_^2−^) ions condensed to form pyrophosphate (P_2_O_7_^4^) with thermal recrystallization and crystal size growth and the formation of tricalcium phosphate was observed, concomitant to the reduction of  P_2_O_7_^4^ ions. The lower amount of carbonate provides less solubility to hydroxyapatite, since carbonate causes crystal defects and does not fit so well in the lattice, generating unstable and more acid-soluble apatite phases. Pyrophosphate is able to inhibit the dissolution of hydroxyapatite crystals, whereas tri- and tetracalcium phosphates are potentially more susceptible to acid dissolution than hydroxyapatite.

The decomposition of the organic matrix is also responsible for increased acid resistance of tooth enamel. The temperature increase in the irradiated surfaces is responsible for the decreased permeability and reducing enamel solubility, causing proteins decomposition. The products of the organic material can obstruct the pores of the tooth enamel, preventing the acid ions penetration [33, 34].

On the other hand, it has been shown that irradiation with laser associated with a topical application of fluoride could increase the acid resistance by increasing the incorporation of fluoride [35] or for the greater transformation of hydroxyapatite in fluorapatite [36]. The synergism between the Nd:YAG laser and fluoride in reducing enamel solubility was verified by Zezell et al. 2009 [6] although Phan et al. 1999 [36] disagree stating that the laser irradiation can induce the formation of fluorapatite by the incorporation of fluoride in the enamel surface layer melted by the temperature increasing.

The temperature rise at pulp chamber in monkeys did not exceed 5.5°C, which would result in permanent/irreversible damage to the pulp [8, 37]. On the other hand, Baldissara et al., 1997 [38], reported that average temperature increases of 11.2°C, seen clinically and histologically, promoted no inflammation in the pulp tissue and thus may have not caused damage to the structure.

The present study compared temperature changes during irradiation with different pulse duration (extra long pulse (10.000 *μ*s), long pulse (700 *μ*s), and short pulse (350 *μ*s)), on specimens of primary enamel using Nd:YAG laser, and no statistically significant difference was observed.

The results of this study showed that the temperature rise on specimens of primary enamel using Nd:YAG laser exceeded 5.5°C. A factor that may have contributed to this thermal change is based on the fact that irradiation was performed at contact mode, without air and water, by means of optical fiber. Similar results were found by Strakas et al., 2103 [39], who verified temperature changes higher than 5.5°C; however, they found significant difference between pulse duration of 180 *μ*s and 320 *μ*s in root canals irradiated with Nd:YAG laser.

Studies proved that when refrigeration is employed during irradiation of dental surfaces, there is a reduction on the temperature increase [40, 41], avoiding, thus, pulp necrosis [42] and carbonization of dentin [41]. The same relation of using water flow to reduce temperature was observed by [26], although they employed Er:YAG laser to irradiate specimens of primary enamel.

The photothermal interaction of Nd:YAG laser with dental tissues is obtained by means of its optical fiber, which, when irradiating dental surface, converts the absorbed laser energy in heat [43], causing higher temperature increase in teeth with less remaining dentin [19, 20, 44, 45], and the Nd:YAG laser wavelength (*λ* 1.064 *μ*m) is poorly absorbed by dental structures [2].

The temperature found in specimens of primary enamel in the present study may differ from the temperature observed at pulp chamber, since, due to the support structures present around the teeth and the blood flow of the pulp tissue, this heat could be dissipated [46, 47]. The pulp temperature increase, related to the use of high power lasers, is based on the amount of energy applied and therefore the exposure time is crucial. High energy densities in short periods of time cause less pulp damage [44], since it is desirable to minimize the heat flux to reduce thermal injuries, providing enough power in less time than the diffusion of heat by conduction through the tissues, considering that the thermal relaxation is inversely proportional to the square of the irradiated volume [46].

The use of specimens of primary enamel with smaller thicknesses in the present study could also provide increased heat generation. This same relation was observed by von Fraunhofer and Allen, 1993 [48], and White et al., 1994 [20], who observed lower temperature changes in structures with greater thicknesses of enamel and dentin.

Thus, the temperature increase found in the present study can be based on the primary teeth mineralization, which is smaller, along with calcium and phosphorus percentage, when compared to permanent teeth [49]; in addition, the thickness of primary enamel is almost half of the permanent tooth enamel [50].

Another factor that may have contributed on the temperature increase in the present study is the thermal conductivity, which is higher in dental enamel than in dentin [16–18]; however, this thermal conductivity exceeds the dentin tissue before reaching the pulp tissue [51]. It has also been stated that anterior primary teeth have the ability to cool more quickly at the beginning of irradiation, followed by a deceleration period. At posterior teeth, the cooling follows a linear decline, due to increased volume in the dental crown and amount of remaining dentin [18].

Comparison of these results with those found in literature is difficult, since the only study published in primary teeth [18] employed different methodology and ablative parameters to evaluate the thermal changes, and in the present study, the method validated in literature was chosen, following the protocols proposed by Contente et al., 2012 [26], and Brandão et al., 2012 [52]. The parameters of irradiation used in this study are recommended in primary enamel and responsible for increased acid resistance to demineralization [4, 6, 27, 28].

## 5. Conclusion

Considering the experimental conditions of this study, it can be concluded that the duration of the laser pulse Nd:YAG increased the temperature of the primary enamel but was not influenced by different pulse durations (extra long pulse (10.000 *μ*s), long pulse (700 *μ*s), and short pulse (350 *μ*s)) used in the irradiation.

## Figures and Tables

**Table 1:** Temperature changes (°C) in specimens of primary enamel, during Nd:YAG laser irradiation.

Types of pulse of Nd:YAG laser	Average ± SD
Short pulse (350 *μ*s)	26.9 ± 12.8
Long pulse (700 *μ*s)	27.3 ± 11.3
Extra long pulse (10.000 *μ*s)	23.1 ± 7.7
